# Dose-response associations of maternal age with pregnancy complications and multimorbidity among nulliparas and multiparas: A multicentric retrospective cohort study in southern China

**DOI:** 10.7189/jogh.13.04117

**Published:** 2023-09-29

**Authors:** Jingya Li, Yamei Li, Yamei Duan, Xiang Xiao, Jiayou Luo, Miyang Luo

**Affiliations:** 1 Department of Women and Children Health, Xiangya School of Public Health, Central South University, Changsha, Hunan Province, China; 2 Department of Social Medicine and Health Management, Xiangya School of Public Health, Central South University, Changsha, Hunan Province, China; 3 Department of Women Health Care, Hunan Provincial Maternal and Child Health Care Hospital, Changsha, Hunan Province, China; 4 Department of Epidemiology, Xiangya School of Public Health, Central South University, Changsha, Hunan Province, China

## Abstract

**Background:**

Advanced maternal age is becoming an increasingly common issue worldwide, presenting substantial health risks to pregnant women. However, dose-response associations of maternal age with a comprehensive range of pregnancy complications and their multimorbidity remain unclear.

**Methods:**

We conducted a retrospective cohort study using data from China’s National Maternal Near Miss Surveillance System for 2017-2018, including 18 hospitals in southern China. We included 135 274 pregnant women aged 15-54 years with a singleton birth. We used multivariable logistic regression and restricted cubic spline to examine dose-response associations between maternal age and various pregnancy complications, as well as multimorbidity. We employed the Apriori algorithm to mine the association rules among pregnancy complications and identify frequent multimorbidity patterns.

**Results:**

We found three distinct patterns of associations between maternal age and specific pregnancy complications. In relation to increasing maternal age, gestational diabetes mellitus, preeclampsia, and gestational hypertension showed nonlinear increasing trends for both nulliparas and multiparas, as did multimorbidity in nulliparas. Conversely, we observed linear increasing trends for placental previa in both nulliparas and multiparas, placental abruption in nulliparas, and multimorbidity in multiparas. Infection and severe anaemia had an approximate J-shaped curve among nulliparas, while postpartum haemorrhage exhibited a similar curve in both nulliparas and multiparas. Advanced maternal age was linked to an elevated risk of multimorbidity during pregnancy or postpartum period, exhibiting more complicated patterns. The most common multimorbidity patterns in this age group were “preeclampsia + gestational diabetes mellitus”, “gestational hypertension + gestational diabetes mellitus”, “infection + gestational diabetes mellitus”, and “placental previa + gestational diabetes mellitus”.

**Conclusions:**

Maternal age was associated with pregnancy complications and multimorbidity in three broad dose-response manners, including approximate J-shaped curves, as well as nonlinear and linear increasing trends, depending on the specific outcome and parity, which may suggest different underlying biological mechanisms. Women with advanced maternal age had a higher risk and more complicated patterns of multimorbidity during pregnancy or postpartum, suggesting that this group should be targeted for more intensive health care.

Childbearing demographics have undergone significant changes in recent decades [1] due to interactions between evolving social and economic factors, as well as advancements in assisted reproductive technology. These changes have led to more women getting pregnant at an advanced maternal age, particularly in high-income countries [2]. Similar trends have been observed in China. According to the latest China Statistical Yearbook, from 2005 to 2015, birth rates increased by 0.3% for women aged 35 to 49 years old, despite an overall decrease in birth rate from 3.4% to 3.1% [3]. However, this demographic shift raises important maternal health concerns.

Advanced maternal age, generally defined as ≥35 years old, has been linked to a wide range of poor pregnancy and perinatal outcomes [4]. Existing studies have reported that advanced maternal age caused increased risks of pregnancy complications, including gestational diabetes mellitus, hypertensive disorders in pregnancy, placental disorders, and postpartum haemorrhage [5-8]. Likewise, very young maternal age (<20 years old) has been found to be associated with increased risks of anaemia and infection [9,10]. Despite existing data on these associations, there is a lack of a comprehensive understanding due to several factors, such as inconsistencies in specific outcomes, small sample sizes, heterogeneity of study designs, lack of controlling for confounders, and failure to account for the modification effect of parity [11-16]. Moreover, prior research has often treated maternal age as a continuous variable within regression models, using only categorisation without assessing linear or nonlinear trends [5-17]. However, it is essential to recognise that these complications may occur to a greater or lesser extent as maternal age changes. Thus, distinguishing how the “response” of distinct pregnancy complications corresponds to the “dose” of maternal age requires further investigation. Furthermore, there have been few studies on the co-occurrence of ≥2 pregnancy complications during a single pregnancy or postpartum period, resulting in insufficient knowledge about the relationship between maternal age and the risk of experiencing multiple concurrent health issues, and prevalent patterns of such multimorbidity. Consequently, several research questions remain on the relationships between maternal age and its potential health repercussions: Are there distinct dose-response relationships between maternal age and the array of pregnancy complications, and how does the multimorbidity of diverse pregnancy complications vary across the spectrum of different maternal age?

Thus, we aimed to conduct a retrospective cohort study in southern China to examine the dose-response associations between maternal age and pregnancy complications, and the related multimorbidity. We also aimed to determine the common patterns of multimorbidity related to pregnancy complications across various maternal age groups among nulliparous and multiparous women. This study may provide valuable insights for both pregnant women and maternal health care providers.

## METHODS

### Study population

We based this retrospective cohort study on data obtained from China’s National Maternal Near Miss Surveillance System for the 2017-2018 period. Details on this surveillance system have been reported elsewhere [18]. We selected 18 hospitals in southern China for analysis, comprising two provincial, three municipal, and 13 county-level hospitals with the capacity to perform obstetric technical operations and with well-established information systems. Each hospital was regionally representative and had over 1000 deliveries annually. The study population was restricted to women aged 15-54 years old who had a singleton birth and complete information for factors and outcomes. We included 135 274 women in this analysis and divided them into three subgroups according to maternal age: <20, 20-34, and ≥35 years old. We obtained ethical approval from the Ethics Committee of Xiangya School of Public Health, Central South University (No. XYGW-2021-103), which waived the requirement for obtaining informed consent due to the retrospective nature of this study. All research procedures followed the approved guidelines and regulations.

### Data collection

Data were prospectively collected within each hospital through a standardised process for all pregnant or postpartum women admitted to the obstetric department. Sociodemographic and obstetric information were recorded and reviewed before discharge by an attending obstetrician or nurse using a unified individual survey form. All data from the completed form were entered into the centralised web-based online reporting system at the National Office for Maternal and Child Health Surveillance of China (NOMCHS). We then extracted data on baseline characteristics and outcomes of interest from this online system by trained staff. Baseline characteristics included maternal age, marital status, educational level, gravidity, times of antenatal examination, parity, history of caesarean section, and level of hospital. Our outcomes of interest were eight types of pregnancy complications, including gestational diabetes mellitus (GDM), gestational hypertension, preeclampsia, postpartum haemorrhage (PPH), placental previa, placental abruption, infection, and severe anaemia.

### Outcome definitions

We diagnosed GDM according to the International Association of Diabetes and Pregnancy Study Group (IADPSG) 2010 criteria [19] and used the International Society for the Study of Hypertension in Pregnancy (ISSHP) 2009 recommendations for the diagnosis of gestational hypertension and preeclampsia [20]. Placental previa was diagnosed by ultrasonography or magnetic resonance imaging showing that the placental margin reached the internal cervical orifice after 28 weeks of gestation. Placental abruption was diagnosed with examinations and positive signs, such as vaginally bleeding and uterine tachysystole, indicating that the placenta partially or completely peeled from the uterine wall after 20 weeks of gestation before delivery. Infections included puerperal infection, abdominal incision infection, urinary tract infection, upper respiratory system infection, and sepsis. We defined severe anaemia as haemoglobin less than 70g/L, and multimorbidity as the co-occurrence of ≥2 pregnancy complications during one pregnancy or postpartum for the same pregnant woman [21].

### Statistical analysis

We described participants’ baseline characteristics using medians with interquartile ranges (IQRs) for non-normal continuous variables and numbers with percentages for categorical variables. We compared the three maternal age groups’ baseline characteristics using a χ^2^ test for categorical variables. The incidence of pregnancy complications and multimorbidity was calculated in each maternal age group stratified by parity (Table S1 in the **Online Supplementary Document**). We established multivariate logistic regression models to obtain adjusted odds ratios (aORs) and 95% confidence intervals (CIs). We modelled maternal age with restricted cubic splines to account for nonlinear relationships with pregnancy complications and multimorbidity. We adjusted for marital status, educational level, gravidity, times of antenatal examination, and level of hospital among nulliparas, and also for history of caesarean section among multiparas. We performed tests for linear trends by entering the median value of each maternal age group as a continuous variable in the models. Statistical tests were two-tailed and *P* < 0.05 was considered statistically significant.

We mined multimorbidity patterns and association rules among pregnancy complications using an Apriori algorithm by setting the minimum support of 0.005 and confidence of 0.02 [22-24] to discern intricate relationships between various pregnancy complications, predicated on their observed patterns of co-occurrence. The minimum support is the threshold frequency below which item sets or rules will be considered infrequent and discarded. We additionally set a minimum support of 0.001 and confidence of 0.001 in the algorithm (Table S2 in the **Online Supplementary Document**).

We defined the *support* of the association rule *A → B* as the frequency of transactions containing both A and B in the whole data set (equation 1):

1) *Support (A → B) = P(A ∩ B) = number of transactions containing both A and B/total number of transactions*

We defined the *confidence* of the association rule *A → B* as the frequency of transactions containing B given the condition of transactions containing *A* in the data set (equation 2):

2) *Confidence (A → B) = P(B | A) = number of transactions containing both A and B/number of transactions containing A*

Lastly, we defined the *lift* of the association rule *A → B* as the ratio of the confidence (*A → B*) to the frequency of transactions containing *B* (equation 3). It is a measure of the direction and strength of the association between *A* and *B* beyond what would be expected by chance. A lift value >1 indicates that *A* and *B* are dependent and correlated positively.

3) *Lift (A → B) = P(A ∩ B)/P(A)P(B)*

We performed statistical analyses using SPSS, version 26.0 (IBM, Armonk, New York, USA) and R, version 4.1.2 (R Core Team, Auckland, New Zealand).

## RESULTS

### Baseline characteristics of the study population according to maternal age groups

**Figure 1:**
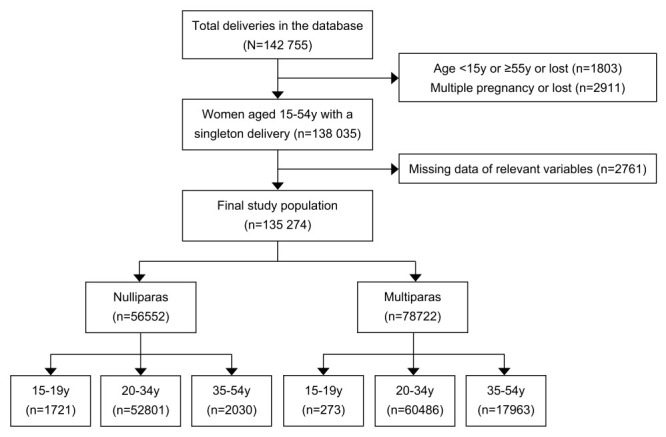
Flowchart of study population.

The cohort comprised 135 274 women aged 15-54 years old with a singleton birth (**Table 1**) and a median maternal age of 29.0 (IQR = 26.0-32.0) years, with 1994 (1.5%), 113 287 (83.7%), and 19 993 (14.8%) subjects in the <20, 20-34 and ≥35-year-old age groups, respectively. Within the cohort, 56 552 (41.8%) women were nulliparas and the other 78 722 (58.2%) women were multiparas. Overall, 99.3% of women were married or cohabiting, 82.5% finished high school education or above, 28.1% of women had never been pregnant before, and 22.9% had a history of caesarean section. Nearly half (49.5%) of women had six to nine antenatal examinations, 31.0% of women had 10 or more, and only 19.6% had five or below. 51.3% of women delivered at county-level, 22.9% at provincial, and 25.8% at municipal hospitals (**Figure 1**).

**Table 1:** Baseline characteristics of the study population according to maternal age groups

		Maternal age groups (y)			
Variables*	**Total (n = 135 274)**	**<20 (n = 1994)**	**20-34 (n = 113287)**	**≥35 (n = 19993)**	***P*-value**
**Maternal age in years, median (IQR)**	29.0 (26.0-32.0)	19.0 (18.0-19.0)	28.0 (26.0-30.0)	37.0 (35.0-39.0)	
**Marital status**					<0.001**†**
Single, divorced or widowed	885 (0.7)	227 (11.4)	581 (0.5)	77 (0.4)	
Married or cohabited	134 389 (99.3)	1767 (88.6)	112 706 (99.5)	19 916 (99.6)	
**Educational level**					<0.001**†**
Elementary or below	728 (0.5)	40 (2.0)	522 (0.5)	166 (0.8)	
Junior high school	22 886 (16.9)	893 (44.8)	19 010 (16.8)	2983 (14.9)	
High school	63 431 (46.9)	915 (45.9)	53 985 (47.7)	8531 (42.7)	
College or above	48 229 (35.7)	146 (7.3)	39 770 (35.1)	8313 (41.6)	
**Gravidity**					<0.001**†**
1	37 942 (28.1)	1360 (68.2)	35 758 (31.6)	824 (4.1)	
2	41 184 (30.4)	480 (24.1)	36 590 (32.3)	4114 (20.6)	
≥3	56 148 (41.5)	154 (7.7)	40 939 (36.1)	15055 (75.3)	
**Times of antenatal examination**					<0.001**†**
≤5	26 440 (19.5)	626 (31.4)	22 086 (19.5)	3728 (18.6)	
6-9	66 941 (49.5)	1097 (55.0)	56 898 (50.2)	8946 (44.7)	
≥10	41 893 (31.0)	271 (13.6)	34 303 (30.3)	7319 (36.6)	
**Parity**					<0.001**†**
Nulliparas	56 552 (41.8)	1721 (86.3)	52 801 (46.6)	2030 (10.2)	
Multiparas	78 722 (58.2)	273 (13.7)	60 486 (53.4)	17 963 (89.8)	
**History of caesarean section**					<0.001**†**
Yes	30 945 (22.9)	41 (2.1)	22 094 (19.5)	8810 (44.1)	
No	104 329 (77.1)	1953 (97.9)	91 193 (80.5)	11183 (55.9)	
**Level of hospital**					<0.001**†**
Provincial	30 976 (22.9)	49 (2.5)	24 376 (21.5)	6551 (32.8)	
Municipal	34 871 (25.8)	304 (15.2)	29 987 (26.5)	4580 (22.9)	
County	69 427 (51.3)	1641 (82.3)	58 924 (52.0)	8862 (44.3)	

IQR – interquartile range

*Values presented as n (%) unless specified differently.

†Statistically significant.

Among the whole population, the incidence of pregnancy complications was as follows (Table S1 in the **Online Supplementary Document**): gestational diabetes mellitus (11.0%), infection (2.5%), preeclampsia (2.1%), gestational hypertension (2.1%), postpartum haemorrhage (1.8%), placental previa (1.1%), placental abruption (0.4%), and severe anaemia (0.4%). Overall, 2.3% of women had ≥2 pregnancy complications during pregnancy or postpartum.

### Analysing associations of maternal age with pregnancy complications and multimorbidity

Among nulliparas, the younger maternal age group of <20 years old was at increased risks of infection (aOR = 1.7) and severe anaemia (aOR = 2.9), yet at a decreased risk of gestational diabetes mellitus (aOR = 0.4); advanced maternal age was associated with increased risks of gestational diabetes mellitus (aOR = 2.1), preeclampsia (aOR = 2.0), gestational hypertension (aOR = 1.9), placental previa (aOR = 3.1), placental abruption (aOR = 1.8), and multimorbidity (aOR = 2.5), compared with the 20-34-year-old age group. Among multiparas, younger maternal age group was at increased risks of placental abruption (aOR = 3.7); advanced maternal age was associated with increased risks of gestational diabetes mellitus (aOR = 2.1), preeclampsia (aOR = 2.5), gestational hypertension (aOR = 2.0), postpartum haemorrhage (aOR = 1.2), placental previa (aOR = 1.7), and multimorbidity (aOR = 2.2) compared with the 20-34-year-old age group (**Table 2**).

**Table 2:** Pregnancy complications and multimorbidity according to maternal age groups, stratified by parity*

	Nulliparas (n = 56 552)				Multiparas (n = 78 722)			
	**<20 years (n = 1721)**		**≥35 years (n = 2030)**		**<20 years (n = 273)**		**≥35 years (n = 17 963)**	
**Outcome**	**OR (95%CI)**	***P*-value**	**OR (95%CI)**	***P*-value**	**OR (95%CI)**	***P*-value**	**OR (95%CI)**	***P*-value**
Gestational diabetes mellitus	0.4 (0.3-0.6)	<0.001†	2.1 (1.9-2.4)	<0.001†	0.5 (0.3-1.1)	0.076	2.1 (2.0-2.2)	<0.001†
Infection	1.7 (1.3-2.2)	<0.001†	1.1 (0.8-1.5)	0.432	1.2 (0.5-2.6)	0.737	1.0 (1.0-1.1)	0.857
Preeclampsia	0.9 (0.6-1.3)	0.583	2.0 (1.6-2.5)	<0.001†	0.6 (0.1-2.4)	0.478	2.5 (2.3-2.8)	<0.001†
Gestational hypertension	0.9 (0.6-1.3)	0.526	1.9 (1.5-2.3)	<0.001†	0.3 (0.0-1.8)	0.177	2.0 (1.7-2.2)	<0.001†
Postpartum haemorrhage	1.4 (0.9-2.0)	0.059	1.2 (0.9-1.5)	0.317	0.7 (0.2-2.2)	0.566	1.2 (1.0-1.3)	0.026†
Placental previa	0.3 (0.1-1.3)	0.107	3.1 (2.3-4.2)	<0.001†	0.6 (0.1-4.2)	0.603	1.7 (1.5-1.9)	<0.001†
Placental abruption	1.0 (0.4-2.4)	0.913	1.8 (1.0-3.2)	0.039†	3.7 (1.2-11.8)	0.028†	1.1 (0.9-1.5)	0.392
Severe anaemia	2.9 (1.8-4.6)	<0.001†	1.5 (0.8-3.0)	0.238	-	-	1.2 (0.9-1.5)	0.230
Multimorbidity	1.1 (0.7-1.6)	0.645	2.5 (2.0-3.1)	<0.001†	0.5 (0.1-2.2)	0.376	2.2 (2.0-2.4)	<0.001†

*Women aged 20-34 years were used as the reference group.

†Statistically significant.

**Figure 2:**
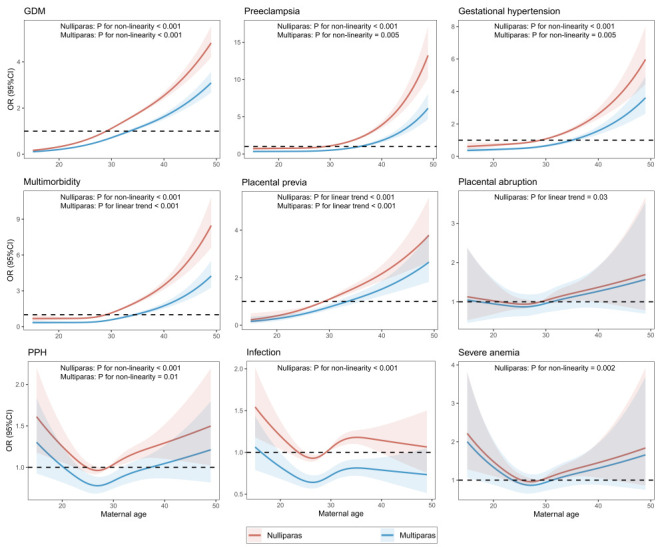
Dose-response associations of maternal age with pregnancy complications and multimorbidity. Solid lines are multivariable adjusted odds ratios, with light bands showing 95% confidence intervals derived from restricted cubic spline regressions with four knots at the 5th, 35th, 65th, and 95th percentiles of maternal age. Reference lines for no association are indicated by the dashed lines at an odds ratio of 1.0. GDM – gestational diabetes mellitus, PPH – postpartum haemorrhage.

As shown in restricted spline curves, we observed nonlinear increasing trends with increasing maternal age for gestational diabetes mellitus, preeclampsia, and gestational hypertension among both nulliparas and multiparas, and multimorbidity among nulliparas (**Figure 2**). As maternal age increased, we observed linear increasing trends for multimorbidity among multiparas, placental previa among both nulliparas and multiparas, and placental abruption among nulliparas. Additionally, the risk of postpartum haemorrhage decreased as the maternal age increased and then showed an upward trend among both nulliparas and multiparas. Similarly, we observed an approximate J-shaped curve for the association between maternal age and infection, as well as severe anaemia among nulliparas. However, we found no evidence of nonlinear or linear associations between maternal age and the risks of placental previa, infection, or severe anaemia among multiparas.

### Describing multimorbidity patterns of pregnancy complications according to maternal age, stratified by parity

In this study, the “support” can be interpreted as the incidence of multimorbidity containing both the former and the latter disease. The “confidence” represents the conditional probability of the latter disease occurring in the presence of the former disease. The “lift” indicates the direction and strength of the association between the former disease and the latter. The larger the “lift” values (>1), the stronger the association between the former disease and the latter disease. Among younger women aged <20 years old, we identified two frequent association rules for nulliparas, showing the most prevalent multimorbidity pattern as “gestational diabetes mellitus + infection”, while the association rule of “infection → gestational diabetes mellitus” with higher confidence. Among elderly women aged ≥35 years old, we identified eight frequent association rules for both nulliparas and multiparas, indicating largely consistent multimorbidity patterns between parities, albeit with varying frequencies. Notably, the most common and closely associated multimorbidity patterns were “gestational diabetes mellitus + preeclampsia”, “gestational hypertension + gestational diabetes mellitus”, “gestational diabetes mellitus + infection”, and “gestational diabetes mellitus + placental previa” irrespective of parity. The top association rule characterised by higher confidence and lift values was “preeclampsia → gestational diabetes mellitus” for nulliparas and “gestational hypertension → gestational diabetes mellitus” for multiparas, respectively (**Table 3**).

**Table 3:** Frequent association rules among pregnancy complications according to maternal age groups, stratified by parity

	Nulliparas					Multiparas				
**Maternal age**	Former*	Latter*	**Support**	**Confidence**	**Lift**	Former*	Latter*	**Support**	**Confidence**	**Lift**
**<20 years**	{GDM}	{Infection}	0.005	0.191	4.919					
	{Infection}	{GDM}	0.005	0.134	4.919					
**≥35 years**	{Preeclampsia}	{GDM}	0.014	0.299	1.331	{Preeclampsia}	{GDM}	0.010	0.261	1.325
	{GDM}	{Preeclampsia}	0.014	0.064	1.331	{GDM}	{Preeclampsia}	0.010	0.051	1.325
	{Gestational hypertension}	{GDM}	0.013	0.299	1.330	{Gestational hypertension}	{GDM}	0.009	0.300	1.525
	{GDM}	{Gestational hypertension}	0.013	0.057	1.330	{GDM}	{Gestational hypertension}	0.009	0.046	1.525
	{Infection}	{GDM}	0.007	0.294	1.309	{Infection}	{GDM}	0.007	0.276	1.404
	{GDM}	{Infection}	0.007	0.033	1.309	{GDM}	{Infection}	0.007	0.036	1.404
	{Placental previa}	{GDM}	0.007	0.246	1.095	{Placental previa}	{GDM}	0.006	0.228	1.159
	{GDM}	{Placental previa}	0.007	0.033	1.095	{GDM}	{Placental previa}	0.006	0.029	1.159

GDM – gestational diabetes mellitus, PPH – postpartum haemorrhage

*The association rule was {A}→{B}, {A} was called the former term of the rule, and {B} was called the latter term of the rule. The minimum support of 0.005 and confidence of 0.02 were set in Apriori algorithm.

## DISCUSSION

In this large, multicentric retrospective study, we systematically investigated dose-response associations of maternal age with a comprehensive range of pregnancy complications and multimorbidity. As maternal age increased, we observed distinct patterns of risk escalation. Specifically, the risk of gestational diabetes mellitus, preeclampsia and gestational hypertension exhibited nonlinear increases among both nulliparas and multiparas, as did the risk of multimorbidity among nulliparas. Conversely, we observed linear increments in the risk of multimorbidity among multiparas, placental previa in both nulliparas and multiparas, and placental abruption among nulliparas. Interestingly, the risk of postpartum haemorrhage initially decreased and then increased among both nulliparas and multiparas, mirroring a J-shaped trend similar to that seen in infection and severe anaemia among nulliparas. We also determined the complex multimorbidity patterns of pregnancy complications across different maternal age groups while accounting for parity. Notably, a prevalent and closely associated multimorbidity pattern involving “gestational diabetes mellitus + infection” emerged among nulliparas aged below 20 years. Among those with advanced maternal age, we found four multimorbidity patterns – “preeclampsia + gestational diabetes mellitus”, “gestational hypertension + gestational diabetes mellitus”, “infection + gestational diabetes mellitus”, and “placental previa + gestational diabetes mellitus” – irrespective of parity. Based on the large sample with standardised records and adjustment for confounding factors, our findings give new insights into the dose-response associations of maternal age with pregnancy complications and multimorbidity, and newly identified multimorbidity patterns during pregnancy or postpartum period by maternal age and parity.

We also observed increased risks in both younger and advanced maternal age groups for several pregnancy complications. Specifically, mothers younger than 20 years old were at increased risks of infection, severe anaemia, and placental abruption, which is consistent with previous studies [9,10]. Mothers with advanced maternal age were at significantly increased risks of most pregnancy complications, including gestational diabetes mellitus, preeclampsia, gestational hypertension, postpartum haemorrhage, placental previa, and placental abruption, which is mostly in line with extensive literature showing that motherhood with advanced maternal age is detrimental to maternal health [5-8]. These increased risks in advanced maternal age could be explained by reproductive ageing, including declined uterine function and damage to the blood and endocrine system [25]. Studies have also shown that older pregnancies are associated with uterine atherosclerosis, impaired uterine myometrial contractility, changes in blood volume, vascular endothelial injury, decreased oxytocin receptors, and insulin receptor dysfunction [26,27].

Although many epidemiologic studies have examined the relationship between categorised maternal age groups and pregnancy complications, dose-response associations of maternal age with pregnancy complications remain unclear. For example, Sheen et al. [17] reported on a bimodal distribution of preeclampsia and postpartum haemorrhage and an increasing trend of gestational diabetes mellitus across maternal age categories. Our findings are consistent with their reported findings of postpartum haemorrhage and gestational diabetes mellitus, except for preeclampsia, which demonstrated a nonlinear increasing trend with maternal age in our study. Another study by Shi et al. [28] showed an inverse J-shaped curve for the association between maternal age and anaemia during pregnancy. In contrast, we found that the association of maternal age with severe anaemia presented an approximate J-shaped curve in nulliparas. Limited sample size at extreme maternal ages and residual confounding might partly explain the discrepancy between our results and those of Shi et al. In the absence of evidence summarising dose-response associations between maternal age and pregnancy complications, we derived three broad patterns of dose-response associations using nonlinear and linear analyses, which may be of use to both women of childbearing age and maternal health care providers. From a preventive perspective of pregnancy complications, although it is generally accepted that giving birth before 35 years old seems ideal [1], our restricted cubic splines suggest the optimal age of childbearing may be before 30 years old due to various increased risks before and after this age.

Here we address a critical gap in the research on maternal health care by exploring multimorbidity patterns of a wide range of pregnancy complications and the association between maternal age and multimorbidity. Our findings suggest that gestational diabetes mellitus is the most common component of multimorbidity patterns, suggesting the importance of addressing abnormal glucose tolerance to prevent the development of other complications. The a priori explanation for this phenomenon is that gestational diabetes mellitus has a high incidence and a relatively long disease duration, which is more likely to be co-morbid with other pregnancy complications. Likewise, the clustering of different diseases into some kind of multimorbidity pattern may be due to some common genetic or environmental pathogenic factors, while mechanisms underlying the development of multimorbidity are complex, interrelated, and diverse, requiring more research [21]. Furthermore, our results of association rules also suggest that preeclampsia, gestational hypertension, infection, and placental previa increased the likelihood of developing gestational diabetes mellitus, possibly providing a basis for further research on disease mechanisms. Additionally, 2.3% of pregnant women had ≥2 pregnancy complications, while a higher risk of multimorbidity is linked to older maternal age. We found that mothers with advanced maternal age were at least two times more likely to undergo ≥2 pregnancy complications compared with mothers aged 20-34 years, and they had more complicated multimorbidity patterns than mothers in other age groups. Our findings support previous studies in other population health fields which showed that age is one of the strong drivers of multimorbidity [21]. These findings highlight the need for a comprehensive approach to maternal health care that considers multimorbidity and its associated disease burden. By identifying the patterns and drivers of multimorbidity, our study provides important insights for health care providers to develop effective prevention and management strategies for pregnant and postpartum women.

Our study has several strengths. First, the high-quality data with a large sample size provided sufficient statistical power to examine associations of interest stratified by parity. Second, we progressively used a methodology of nonlinear and linear analyses to determine dose-response associations of maternal age with a comprehensive range of pregnancy complications. Third, this study is the first to show multimorbidity patterns of pregnancy complications and the association of multimorbidity with maternal age. To the best of our knowledge, no study has yet investigated multimorbidity during pregnancy or postpartum period. This study also has several limitations. First, it was conducted in southern China, which may limit the generalisability to other regions of China. Second, data for potentially strong confounding factors such as family planning methods and socioeconomic status were not collected [29], so we cannot rule out potential confounding by these or other unknown factors. Third, despite the large sample size, the small number of cases at extreme maternal ages may lead to bias in risk estimates – for example, a wide range of confidence intervals.

## CONCLUSIONS

We further determined the nuanced relationships between maternal age and pregnancy complications and multimorbidity, revealing three broad dose-response manners: approximate J-shaped curves, nonlinear and increasing linear trends for different outcomes, with stronger associations in nulliparas than in multiparas. Women with advanced maternal age faced a heightened risk and more complicated patterns of multimorbidity during pregnancy or the postpartum period. Our findings could help with providing age-specific maternal health recommendations for high-risk groups and identifying major multimorbidity patterns for prevention and control in practice.

## Additional material


Online Supplementary Document
