# Supplementary material for: Dose-response associations of maternal age with pregnancy complications and multimorbidity among nulliparas and multiparas: A multicentric retrospective cohort study in southern China
Source: J Glob Health. 2023 Sep 29;13:04117. doi: 10.7189/jogh.13.04117 (PMC10535007; doi:10.7189/jogh.13.04117)
Supplement: Online Supplementary Document [file jogh-13-04117-s001.pdf]

Table S1. Incidence of pregnancy complications and multimorbidity according to maternal age groups, stratified by parity

Outcome N (%)	Total (n=135274)	Nulliparas (n=56552)			Multiparas (n=78653)		
		<20y (n=1721)	20-34y (n=52801)	≥35y (n=2030)	<20y (n=273)	20-34y (n=60486)	≥35y (n=17963)
Gestational diabetes mellitus	14875 (11.0%)	47 (2.7%)	5358 (10.1%)	456 (22.5%)	9 (3.3%)	5468 (9.0%)	3537 (19.7%)
Infectious diseases	3377 (2.5%)	67 (3.9%)	1289 (2.4%)	51 (2.5%)	6 (2.2%)	1501 (2.5%)	463 (2.6%)
Preeclampsia	2830 (2.1%)	30 (1.7%)	1152 (2.2%)	97 (4.8%)	2 (0.7%)	863 (1.4%)	686 (3.8%)
Gestational hypertension	2778 (2.1%)	31 (1.8%)	1191 (2.3%)	87 (4.3%)	1 (0.4%)	922 (1.5%)	546 (3.0%)
Postpartum hemorrhage	2499 (1.8%)	34 (2.0%)	1070 (2.0%)	53 (2.6%)	3 (1.1%)	1001 (1.7%)	338 (1.9%)
Placental previa	1497 (1.1%)	2 (0.1%)	327 (0.6%)	61 (3.0%)	1 (0.4%)	659 (1.1%)	447 (2.5%)
Placental abruption	517 (0.4%)	5 (0.3%)	189 (0.4%)	14 (0.7%)	3 (1.1%)	221 (0.4%)	85 (0.5%)
Severe anemia	584 (0.4%)	24 (1.4%)	188 (0.4%)	9 (0.4%)	0 (0.0%)	279 (0.5%)	84 (0.5%)
Multimorbidity	3109 (2.3%)	29 (1.7%)	1071 (2.0%)	119 (5.9%)	2 (0.7%)	1076 (1.8%)	812 (4.5%)

Table S2. Frequent association rules among pregnancy complications according to maternal age groups, stratified by parity

Maternal age	Nulliparas					Multiparas				
	Former*	Latter*	Support	Confidence	Lift	Former*	Latter*	Support	Confidence	Lift
**<20y**	{GDM}	{Infection}	0.005	0.191	4.919	{GDM}	{PPH}	0.004	0.111	10.111
	{Infection}	{GDM}	0.005	0.134	4.919	{Placental abruption}	{Infection}	0.004	0.333	15.167
	{Severe anemia}	{PPH}	0.003	0.250	12.654	{Infection}	{Placental abruption}	0.004	0.167	15.167
	{PPH}	{Severe anemia}	0.003	0.176	12.654					
	{Preeclampsia}	{Infection}	0.002	0.100	2.569					
	{Infection}	{Preeclampsia}	0.002	0.045	2.569					
	{Gestational hypertension}	{Infection}	0.001	0.065	1.657					
	{Infection}	{Gestational hypertension}	0.001	0.030	1.657					
	{PPH}	{Infection}	0.001	0.059	1.511					
	{Infection}	{PPH}	0.001	0.030	1.511					
**20-34y**	{Gestational hypertension}	{GDM}	0.004	0.196	1.928	{Infection}	{GDM}	0.003	0.137	1.518
	{GDM}	{Gestational hypertension}	0.004	0.043	1.928	{GDM}	{Infection}	0.003	0.038	1.518
	{Preeclampsia}	{GDM}	0.004	0.194	1.916	{Gestational hypertension}	{GDM}	0.003	0.188	2.076
	{GDM}	{Preeclampsia}	0.004	0.042	1.916	{GDM}	{Gestational hypertension}	0.003	0.032	2.076
	{Infection}	{GDM}	0.003	0.119	1.170	{Preeclampsia}	{GDM}	0.003	0.198	2.192
	{GDM}	{Infection}	0.003	0.029	1.170	{GDM}	{Preeclampsia}	0.003	0.031	2.192
	{GDM}	{PPH}	0.002	0.022	1.068	{GDM}	{PPH}	0.002	0.017	1.039
	{Severe anemia}	{PPH}	0.001	0.404	19.949	{Placental previa}	{GDM}	0.001	0.126	1.393
	{PPH}	{Severe anemia}	0.001	0.071	19.949	{GDM}	{Placental previa}	0.001	0.015	1.393
	{Placental previa}	{GDM}	0.001	0.183	1.808	{Severe anemia}	{PPH}	0.001	0.258	15.594
	{GDM}	{Placental previa}	0.001	0.011	1.808	{PPH}	{Severe anemia}	0.001	0.072	15.594
	{Preeclampsia}	{Infection}	0.001	0.051	2.098					
	{Infection}	{Preeclampsia}	0.001	0.046	2.098					
**≥35y**	{Preeclampsia}	{GDM}	0.014	0.299	1.331	{Preeclampsia}	{GDM}	0.010	0.261	1.325
	{GDM}	{Preeclampsia}	0.014	0.064	1.331	{GDM}	{Preeclampsia}	0.010	0.051	1.325
	{Gestational hypertension}	{GDM}	0.013	0.299	1.330	{Gestational hypertension}	{GDM}	0.009	0.300	1.525
	{GDM}	{Gestational hypertension}	0.013	0.057	1.330	{GDM}	{Gestational hypertension}	0.009	0.046	1.525
	{Infection}	{GDM}	0.007	0.294	1.309	{Infection}	{GDM}	0.007	0.276	1.404
	{GDM}	{Infection}	0.007	0.033	1.309	{GDM}	{Infection}	0.007	0.036	1.404
	{Placental previa}	{GDM}	0.007	0.246	1.095	{Placental previa}	{GDM}	0.006	0.228	1.159
	{GDM}	{Placental previa}	0.007	0.033	1.095	{GDM}	{Placental previa}	0.006	0.029	1.159
	{GDM}	{PPH}	0.005	0.024	0.924	{GDM}	{PPH}	0.004	0.019	0.992
	{Infection}	{Preeclampsia}	0.002	0.098	2.052	{Infection}	{Preeclampsia}	0.003	0.106	2.771
	{Preeclampsia}	{Infection}	0.002	0.052	2.052	{Preeclampsia}	{Infection}	0.003	0.071	2.771
	{Placental previa}	{PPH}	0.002	0.082	3.139	{Placental previa}	{Infection}	0.002	0.069	2.691
	{Placental abruption}	{GDM}	0.002	0.286	1.272	{Infection}	{Placental previa}	0.002	0.067	2.691
	{GDM}	{Placental abruption}	0.002	0.009	1.272	{Placental previa}	{PPH}	0.002	0.060	3.210
	{Severe anemia}	{PPH}	0.001	0.333	12.767	{Infection}	{Gestational hypertension}	0.002	0.058	1.919
	{PPH}	{Severe anemia}	0.001	0.057	12.767	{Gestational hypertension}	{Infection}	0.002	0.049	1.919
	{Infection}	{Gestational hypertension}	0.001	0.059	1.373	{Severe anemia}	{PPH}	0.001	0.298	15.817
	{Gestational hypertension}	{Infection}	0.001	0.034	1.373	{PPH}	{Severe anemia}	0.001	0.074	15.817
	{Placental previa}	{Preeclampsia}	0.001	0.049	1.029	{Placental abruption}	{GDM}	0.001	0.259	1.314
	{Preeclampsia}	{Placental previa}	0.001	0.031	1.029	{GDM}	{Placental abruption}	0.001	0.006	1.314

GDM = gestational diabetes mellitus; PPH = postpartum hemorrhage.

*The association rule was {A}⇒{B}, {A} was called the former term of the rule, and {B} was called the latter term of the rule. The minimum support of 0.001 and confidence of 0.001 were set in Apriori algorithm.
